# Validation of Modified Albumin‐Bilirubin‐Tumor Stage as a Prognostic Model in Early‐Stage Hepatocellular Carcinoma Treated With Radiofrequency Ablation

**DOI:** 10.1002/kjm2.70313

**Published:** 2026-11-02

**Authors:** Cheng‐Ying Hsieh, Wen‐Shuo Yeh, Yi‐Hao Yen, Yuan‐Hung Kuo, Ming‐Chao Tsai, Sheng‐Nan Lu, Chao‐Hung Hung, Jing‐Houng Wang, Chien‐Hung Chen, Kwong‐Ming Kee

**Affiliations:** ^1^ Division of Hepatogastroenterology, Department of Internal Medicine Kaohsiung Chang Gung Memorial Hospital, and Chang Gung University College of Medicine Kaohsiung City Taiwan; ^2^ Division of Gastroenterology and Hepatology, E‐Da Dachang Hospital I‐Shou University Kaohsiung City Taiwan; ^3^ Division of Gastroenterology and Hepatology, Department of Internal Medicine Jen‐Ai Hospital Taichung City Taiwan

**Keywords:** hepatic reserve, prognostic model, recurrence‐free survival, tumor stage

## Abstract

Survival after radiofrequency ablation (RFA) for very early‐ or early‐stage hepatocellular carcinoma (HCC) remains heterogeneous because prognosis depends on both tumor burden and hepatic reserve. We retrospectively evaluated treatment‐naive patients with Barcelona Clinic Liver Cancer (BCLC) Stages 0 or A HCC treated with RFA as initial therapy from January 2002 to September 2016. Patients with Child‐Pugh class A or B and complete ablation were included. Four prespecified total scores, Child‐Pugh‐tumor (CP‐T), albumin‐bilirubin‐tumor (ALBI‐T), modified ALBI‐tumor (mALBI‐T), and platelet‐ALBI‐tumor (pALBI‐T) were compared using the Akaike information criterion (AIC), Harrell concordance index (C‐index), time‐dependent area under the receiver operating characteristic curve, calibration, and subgroup analyses. Of 1238 patients screened, 950 were included; 831 (87.5%) were Child‐Pugh class A. During a median follow‐up of 5.44 years, 417 deaths occurred; 5‐ and 10‐year overall survival rates were 58.0% and 35.8%, respectively. The American Joint Committee on Cancer eighth‐edition T stage illustrated the best tumor‐stage performance and was used in the total scores, while mALBI‐T had the lowest AIC (5052.2) and highest Harrell C‐index (0.628; 95% confidence interval, 0.601–0.655). Paired bootstrap differences versus CP‐T, ALBI‐T, and pALBI‐T were statistically significant, but modest, while mALBI‐T performed best at 5 years and in Child‐Pugh class A patients, whereas all models showed limited discrimination in Child‐Pugh class B patients. Apparent calibration was generally close, and mALBI‐T also had the best fit for recurrence‐free survival. mALBI‐T provides a simple prognostic model for long‐term survival stratification after complete ablation in early‐stage hepatocellular carcinoma, especially in Child‐Pugh class A patients.

## 1 Introduction

Hepatocellular carcinoma (HCC) is the most common primary liver cancer [1]. Liver cancer was the third leading cause of cancer‐related death globally in 2022 [1], while liver and intrahepatic bile duct cancer ranked second in Taiwan in 2025 [2]. Unlike most solid tumors, HCC usually develops in a chronically diseased liver; survival is accordingly determined by both tumor burden and hepatic reserve [3] while accurate prognostic assessment should evaluate both components.

This issue is especially relevant in patients with very early‐ (Barcelona Clinic Liver Cancer [BCLC] Stage 0) or early‐stage (BCLC Stage A) HCC. Surveillance supports diagnosis at potentially curative stages, while antiviral therapy can help preserve hepatic reserve [3]. The BCLC staging system is widely used for HCC treatment allocation because it integrates tumor burden, liver function, and performance status [4]. For BCLC stages 0 or A HCC, curative‐intent therapies, including resection, transplantation, and locoregional ablation, are recommended in contemporary treatment guidelines [4, 5, 6, 7, 8] with radiofrequency ablation (RFA) being commonly used for selected patients with small HCC [9, 10]. However, complete local ablation does not eliminate long‐term prognostic heterogeneity, where in this setting, differences in hepatic reserve may influence decompensation, tolerance of salvage therapy, and long‐term survival.

Poor hepatic reserve is associated with worse overall survival (OS) after RFA [11, 12, 13], making liver‐function assessment important for both ablation safety and prognosis [14, 15, 16, 17]. Liver function in HCC has traditionally been graded using the Child‐Pugh classification [18], but this relies partly on subjective variables including ascites and hepatic encephalopathy while using broad categories with empirically defined cutoffs [19]. The albumin‐bilirubin (ALBI) grade was developed as an objective measure based only on serum albumin and total bilirubin, with prognostic performance comparable to Child‐Pugh without subjective variables [19]. Although ALBI grade has been widely validated [20], ALBI Grade 2 remains heterogeneous. Modified ALBI (mALBI) grade subdivides Grade 2 into Grades 2a and 2b using an additional cutoff of −2.27 derived from indocyanine green retention data, yielding four Grades: 1, 2a, 2b, and 3 [21, 22]. Platelet‐ALBI (pALBI) grade additionally incorporates platelet count as a surrogate marker of portal hypertension and hepatic fibrosis [23].

Because prognosis reflects both the liver and the tumor, these hepatic reserve grades can be combined with the tumor stage to create total scoring systems. Patients with BCLC Stages 0 or A HCC differ in tumor size and number, which hepatic‐reserve grades do not capture [4]. Total scoring systems also facilitate comparisons across institutions and treatment modalities. The Okuda staging system was the first widely used model to combine tumor burden and hepatic function, followed by CLIP, JIS, Tokyo score, bm‐JIS, modified JIS, and later ALBI‐based systems such as ALBI‐T and mALBI‐T [24, 25]. Adding the tumor stage to ALBI or mALBI grades produces ALBI‐T and mALBI‐T scores, analogous to the Japan Integrated Staging score that pairs Child‐Pugh class with tumor‐node‐metastasis stage.

In a large multicenter Japanese study, mALBI‐T predicted survival better than ALBI‐T and the Japan Integrated Staging score [22], with subsequent validation in an Egyptian cohort [26]. However, these studies included mixed tumor stages and treatment modalities and used the Liver Cancer Study Group of Japan tumor‐node‐metastasis system. Whether mALBI‐T retains its advantage in BCLC Stages 0 or A HCC after complete RFA has not been fully established.

We therefore conducted this study to validate and compare four combined hepatic reserve and tumor‐stage scores, including Child‐Pugh‐tumor (CP‐T), ALBI‐T, mALBI‐T, and pALBI‐T for predicting OS in a large single‐center cohort of patients with BCLC Stages 0 or A HCC who received RFA as initial therapy and achieved complete ablation. To optimize the tumor component, four candidate tumor‐staging systems were compared before construction of the total scores. Model performance was further assessed by calibration, clinically relevant subgroup analyses, and recurrence‐free survival as a secondary endpoint.

## 2 Methods

### 2.1 Patients

This retrospective cohort study screened 1238 patients with treatment‐naive HCC who received RFA as initial therapy and achieved complete radiologic ablation at Kaohsiung Chang Gung Memorial Hospital from January 2002 through September 2016. HCC was diagnosed according to the American Association for the Study of Liver Diseases guidance [6]. Patients with BCLC stages B, C, or D, loss to follow‐up, or incomplete baseline laboratory data were excluded. The final cohort comprised 950 patients with BCLC Stages 0 or A HCC and Child‐Pugh classes A or B liver function (Figure 1; Table 1). A total of 288 patients were excluded, but the number excluded for each reason was unavailable.

**FIGURE 1:**
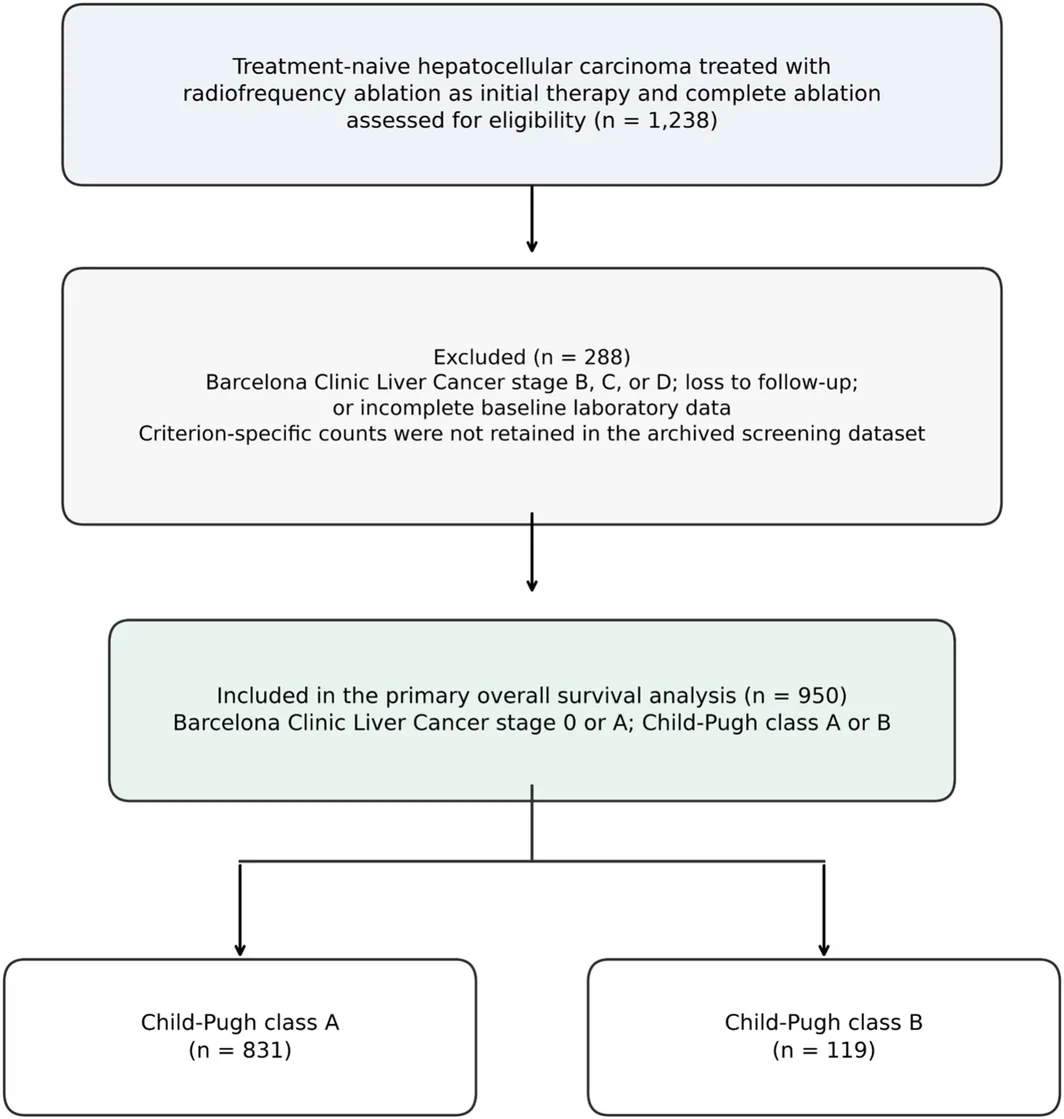
Study flow diagram. Of 1238 patients screened, 288 were excluded and 950 were included. Criterion‐specific exclusion counts were not retained in the archived dataset. BCLC, Barcelona Clinic Liver Cancer; HCC, hepatocellular carcinoma; RFA, radiofrequency ablation.

**TABLE 1:** Baseline characteristics of patients with hepatocellular carcinoma (*N* = 950).

Characteristic	Value
Age, years, mean ± SD	64.2 ± 10.3
Male/female sex, *n* (%)	591 (62.2)/359 (37.8)
Performance status 0/1, *n* (%)	892 (93.9)/58 (6.1)
HCV alone, *n* (%)	450 (47.4)
HBV alone, *n* (%)	326 (34.3)
HBV/HCV coinfection, *n* (%)	67 (7.1)
Non‐HBV non‐HCV, *n* (%)	107 (11.3)
Child‐Pugh class A/B, *n* (%)	831 (87.5)/119 (12.5)
BCLC Stage 0/A, *n* (%)	323 (34.0)/627 (66.0)
Tumor number 1/2/3, *n* (%)	749 (78.8)/162 (17.1)/39 (4.1)
Tumor size, cm, mean ± SD	2.22 ± 0.74
AST, U/L, mean ± SD	64.4 ± 44.7
ALT, U/L, mean ± SD	60.5 ± 48.6
Prothrombin time, sec, mean ± SD	11.1 ± 0.94
INR, mean ± SD	1.06 ± 0.09
Platelets, 10^4^/μL, mean ± SD	13.1 ± 6.14
Albumin, g/dL, mean ± SD	3.78 ± 0.59
AFP ≤ 20/> 20 ng/mL, *n* (%)	561 (59.1)/389 (40.9)
Total bilirubin, mg/dL, mean ± SD	1.03 ± 0.56
AJCC 8th edition T Stage T1a/T1b/T2, *n* (%)	363 (38.2)/386 (40.6)/201 (21.2)
ALBI Grade 1/2/3, *n* (%)	400 (42.1)/500 (52.6)/50 (5.3)
mALBI Grade 1/2a/2b/3, *n* (%)	400 (42.1)/207 (21.8)/293 (30.8)/50 (5.3)
pALBI Grade 1/2/3, *n* (%)	451 (47.5)/371 (39.1)/128 (13.5)

*Note:* Non‐HBV non‐HCV indicates negative serologic evidence for both HBV and HCV infection.

Abbreviations: AFP, alpha‐fetoprotein; AJCC, American Joint Committee on Cancer; ALBI, albumin‐bilirubin; ALT, alanine aminotransferase; AST, aspartate aminotransferase; BCLC, Barcelona Clinic Liver Cancer; HBV, hepatitis B virus; HCV, hepatitis C virus; INR, international normalized ratio; mALBI, modified albumin‐bilirubin; pALBI, platelet‐albumin‐bilirubin.

The Institutional Review Board of Kaohsiung Chang Gung Memorial Hospital approved the study (201801813B0; November 23, 2018) and waived informed consent because of its retrospective design while complying with the Declaration of Helsinki. Follow‐up ended in December 2018.

### 2.2 Assessment of Hepatic Reserve and Tumor Stage

Clinical and laboratory data were obtained from patient records. ALBI score was −0.085 × albumin + 0.66 × log_10_ bilirubin (albumin, g/L; bilirubin, micromol/L). ALBI grades were 1 (≤ −2.60), 2 (> −2.60 to ≤ −1.39), and 3 (> −1.39) [19]. ALBI Grade 2 was subdivided into mALBI grade 2a (> −2.60 to < −2.27) and 2b (≥ −2.27 to ≤ −1.39) [21, 22]. pALBI score was 2.02 × log_10_ bilirubin—0.37 × (log_10_ bilirubin)^2^–0.04 × albumin—3.48 × log_10_ platelets + 1.01 × (log_10_ platelets)^2^, with platelets in 10^9^/L. pALBI grades were 1 (≤ −2.53), 2 (> −2.53 to ≤ −2.09), and 3 (> −2.09) [23].

Candidate systems included BCLC tumor‐burden categories, sixth edition Liver Cancer Study Group of Japan staging, and seventh and eighth edition American Joint Committee on Cancer (AJCC) T stage. The system with the lowest Akaike information criterion (AIC) and highest Harrell concordance index (C‐index) for OS was selected.

CP‐T, ALBI‐T, mALBI‐T, and pALBI‐T summed tumor‐stage and hepatic‐reserve points (Table 2). Child‐Pugh classes A, B, and C, and ALBI and pALBI Grades 1, 2, and 3 received 0, 1, and 2 points respectively; mALBI Grades 1, 2a, 2b, and 3 received 0, 1, 2, and 3 points.

**TABLE 2:** Scoring system for total hepatic reserve and tumor‐stage models.

Component	Score 0	Score 1	Score 2	Score 3
AJCC 8th edition T stage	T1a	T1b	T2	—
Child‐Pugh class	A	B	C	—
ALBI grade	1	2	3	—
mALBI grade	1	2a	2b	3
pALBI grade	1	2	3	—

*Note:* Each total score is the sum of the hepatic‐reserve and tumor‐stage points shown above. AJCC 8th edition T‐stage categories represented in this cohort: T1a, single tumor ≤ 2 cm; T1b, single tumor > 2 cm without vascular invasion; T2, multiple tumors, none > 5 cm, without vascular invasion.

Abbreviations: AJCC, American Joint Committee on Cancer; ALBI, albumin‐bilirubin; mALBI, modified albumin‐bilirubin; pALBI, platelet‐albumin‐bilirubin.

### 2.3 RFA And Follow‐Up

RFA was performed under real‐time ultrasonographic guidance by hepatologists with more than 5 years of procedural experience. Systems used during the study period included Cool‐tip RF (Medtronic, Minneapolis, Minnesota, USA), Big‐tip RF (RF Medical, Seoul, Korea), and Viva RF (STARmed, Seoul, Republic of Korea). The energy‐deposition algorithm for each system followed the manufacturer's instructions. The device used for each procedure was not retained in the archived analytic dataset. Contrast‐enhanced computed tomography or magnetic resonance imaging was performed approximately 1 month later. Complete ablation was defined as absence of enhancement in the ablation zone. Patients were followed using alpha‐fetoprotein, liver‐function tests, ultrasonography, and dynamic imaging.

### 2.4 Outcomes and Statistical Analysis

The primary endpoint was OS from baseline HCC diagnosis to death from any cause; surviving patients were censored at last follow‐up. Median follow‐up was estimated by reverse Kaplan–Meier analysis, while recurrence‐free survival (RFS), analyzed secondarily, was measured from initial RFA to first recurrence or death. First salvage treatment after recurrence was summarized.

Each score was fitted categorically in a Cox model to estimate AIC and survival probabilities. Lower AIC indicated better fit. Harrell C‐indices were calculated from the ordinal scores, with percentile 95% confidence intervals (CIs) and paired differences estimated using 2000 patient‐level bootstrap samples. Inverse‐probability‐of‐censoring‐weighted time‐dependent areas under the receiver operating characteristic curve (AUROCs) were estimated at 1, 5, and 10 years with 800 bootstrap samples. Apparent calibration compared Cox‐predicted survival with Kaplan–Meier survival within score categories in the same cohort; inverse‐probability‐of‐censoring‐weighted Brier scores quantified prediction error.

Child‐Pugh subgroup analyses reported AIC per patient (AIC/N), with model fit compared within each subgroup. Sensitivity analyses examined diagnosis era (2002–2009 vs. 2010–2016), sex, and liver‐disease etiology using Cox score‐by‐subgroup interaction tests.

Exploratory complete‐case analyses compared mALBI‐T with prognostic nutritional index (PNI), geriatric nutritional risk index (GNRI), and neutrophil‐to‐lymphocyte ratio (NLR). Lymphocyte‐to‐monocyte ratio could not be calculated because monocyte counts were unavailable. A model using the five dichotomous variables reported by Kao et al. (age > 65 years, international normalized ratio > 1.1, alpha‐fetoprotein > 20 ng/mL, multiple tumors, and ALBI Grade 2/3) was refitted locally for comparison [27].

Core score components, OS, and RFS data were complete for all 950 patients, and no imputation was performed, although one recurrence indicator conflicting with a documented recurrence date was adjudicated as recurrent before analysis. Biomarker comparisons used 756 common complete cases, excluding 194 patients with missing inputs. Analyses used Python 3.12.13, lifelines 0.30.3, and scikit‐survival 0.28.0. Two‐sided *p* < 0.05 was significant.

## 3 Results

### 3.1 Patient Characteristics and Follow‐Up

The flow of patients is shown in Figure 1, and baseline characteristics are summarized in Table 1. Mean age was 64.2 ± 10.3 years, 591 patients (62.2%) were male, and 831 (87.5%) were Child‐Pugh class A. A solitary tumor was present in 749 patients (78.8%), and mean tumor size was 2.22 ± 0.74 cm. ALBI Grade 2 included 500 patients and was divided into mALBI Grades 2a in 207 and 2b in 293. During a median follow‐up of 5.44 years (95% CI, 5.19–5.72), 417 patients died. The 1‐, 3‐, 5‐, and 10‐year OS rates were 94.6%, 73.6%, 58.0%, and 35.8%, respectively.

### 3.2 Selection of Tumor Stage

AJCC eighth edition T stage had the lowest AIC (5130.746) and highest Harrell C‐index (0.552; 95% CI, 0.524–0.581), compared with sixth edition Liver Cancer Study Group of Japan stage (AIC 5131.585; C‐index 0.542; 95% CI, 0.514–0.568), BCLC tumor‐burden categories (5142.348; 0.525; 95% CI, 0.502–0.547), and AJCC seventh edition T stage (5143.961; 0.526; 95% CI, 0.504–0.548). It was therefore used in the total scores (Table 2).

### 3.3 Overall Survival Performance and Calibration

Higher scores were associated with shorter OS for each model (Figure 2). mALBI‐T had the lowest AIC (5052.2) and highest Harrell C‐index (0.628; 95% CI, 0.601–0.655) (Table 3 and Figure 3). Its C‐index was higher than CP‐T by 0.050 (95% CI, 0.031–0.070; *p* < 0.001), ALBI‐T by 0.021 (95% CI, 0.010–0.033; *p* < 0.001), and pALBI‐T by 0.019 (95% CI, 0.004–0.034; *p* = 0.011).

**FIGURE 2:**
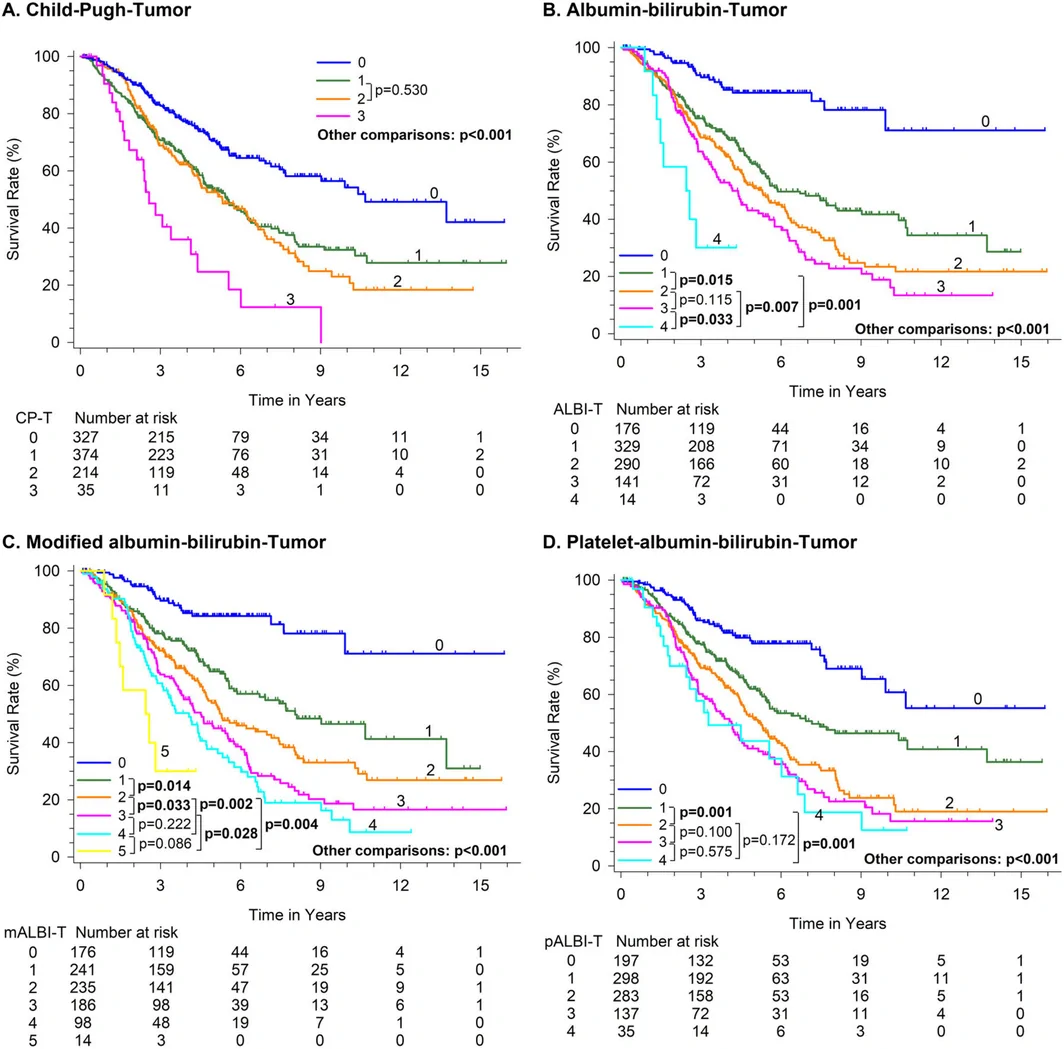
Kaplan–Meier overall survival according to total scoring systems. Survival was stratified by (A) Child‐Pugh‐tumor, (B) albumin‐bilirubin‐tumor, (C) modified albumin‐bilirubin‐tumor, and (D) platelet‐albumin‐bilirubin‐tumor scores. ALBI‐T, albumin‐bilirubin‐tumor; CP‐T, Child‐Pugh‐tumor; mALBI‐T, modified albumin‐bilirubin‐tumor; pALBI‐T, platelet‐albumin‐bilirubin‐tumor.

**TABLE 3:** Overall survival performance and paired comparison of total scoring systems.

Model	AIC	Delta AIC	Harrell C‐index (95% CI)	mALBI‐T minus comparator (95% CI)	*p*
CP‐T	5106.0	53.8	0.577 (0.551–0.605)	0.050 (0.031–0.070)	< 0.001
ALBI‐T	5068.9	16.7	0.606 (0.579–0.634)	0.021 (0.010–0.033)	< 0.001
mALBI‐T	5052.2	0.0	0.628 (0.601–0.655)	Reference	—
pALBI‐T	5083.6	31.4	0.609 (0.581–0.637)	0.019 (0.004–0.034)	0.011

*Note:* Positive paired differences indicate higher discrimination for mALBI‐T.

Abbreviations: AIC, Akaike information criterion; ALBI‐T, albumin‐bilirubin‐tumor; CI, confidence interval; CP‐T, Child‐Pugh‐tumor; mALBI‐T, modified albumin‐bilirubin‐tumor; pALBI‐T, platelet‐albumin‐bilirubin‐tumor.

**FIGURE 3:**
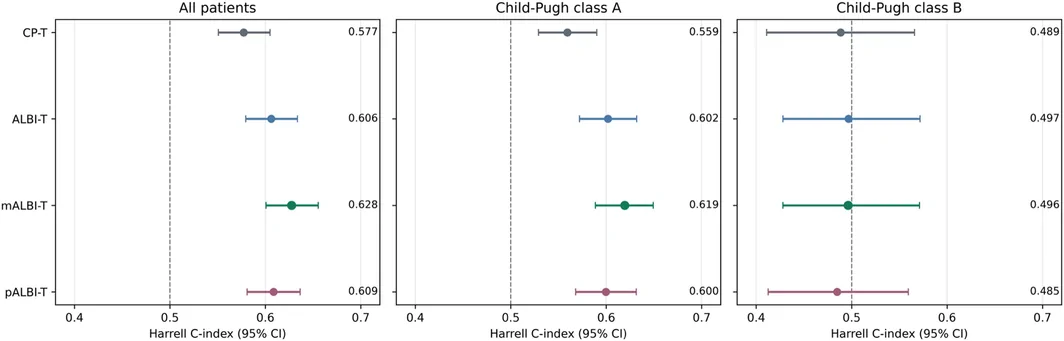
Harrell C‐indices for overall survival in all patients and Child‐Pugh class subgroups. Points show Harrell C‐indices and bars show 95% bootstrap confidence intervals. ALBI‐T, albumin‐bilirubin‐tumor; CI, confidence interval; CP‐T, Child‐Pugh‐tumor; mALBI‐T, modified albumin‐bilirubin‐tumor; pALBI‐T, platelet‐albumin‐bilirubin‐tumor.

pALBI‐T had the highest 1‐year AUROC (0.648), whereas mALBI‐T had the highest 5‐year AUROC (0.648) (Table 4). At 10 years, mALBI‐T and pALBI‐T were similar (0.649 for both; difference, −0.001; *p* = 0.965). Apparent calibration of mALBI‐T was generally close at 1, 5, and 10 years, although uncertainty increased in sparsely represented high‐risk categories (Figure 4). Its Brier scores were 0.050, 0.226, and 0.219, respectively. Calibration for all models is shown in Supplementary Figure 1.

**TABLE 4:** Time‐dependent discrimination and prediction error for overall survival.

Model	1‐year AUROC (95% CI)	5‐year AUROC (95% CI)	10‐year AUROC (95% CI)	1‐year Brier	5‐year Brier	10‐year Brier
CP‐T	0.556 (0.491–0.625)	0.588 (0.547–0.629)	0.634 (0.561–0.714)	0.051	0.235	0.216
ALBI‐T	0.605 (0.539–0.670)	0.621 (0.581–0.664)	0.627 (0.551–0.709)	0.050	0.229	0.221
mALBI‐T	0.632 (0.562–0.696)	0.648 (0.607–0.693)	0.649 (0.572–0.728)	0.050	0.226	0.219
pALBI‐T	0.648 (0.585–0.711)	0.616 (0.572–0.662)	0.649 (0.571–0.730)	0.050	0.234	0.216

*Note:* AUROC values were inverse‐probability‐of‐censoring weighted. Lower Brier scores indicate lower prediction error.

Abbreviations: ALBI‐T, albumin‐bilirubin‐tumor; AUROC, area under the receiver operating characteristic curve; CI, confidence interval; CP‐T, Child‐Pugh‐tumor; mALBI‐T, modified albumin‐bilirubin‐tumor; pALBI‐T, platelet‐albumin‐bilirubin‐tumor.

**FIGURE 4:**
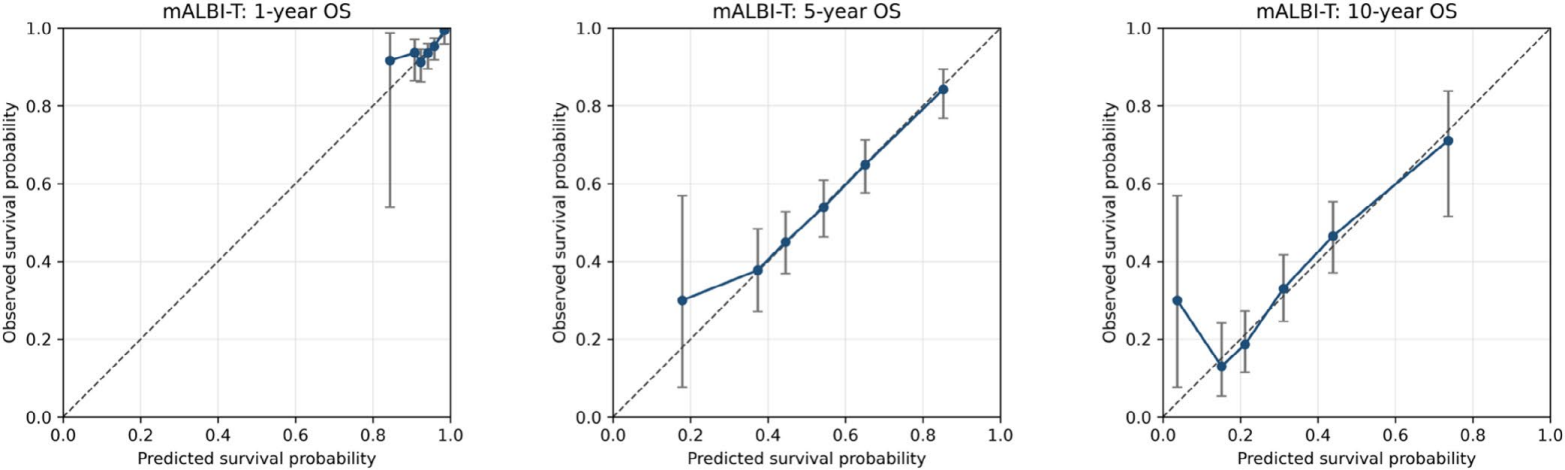
Apparent calibration of modified albumin‐bilirubin‐tumor score. Cox‐predicted and Kaplan–Meier‐observed survival probabilities are shown at 1, 5, and 10 years by score category. Bars show 95% confidence intervals for observed survival. mALBI‐T, modified albumin‐bilirubin‐tumor; OS, overall survival.

### 3.4 Subgroup and Sensitivity Analyses

In Child‐Pugh class A patients, mALBI‐T had the lowest AIC/N (4.84) and highest C‐index (0.619; 95% CI, 0.589–0.649) (Table 5 and Figure 3). In Child‐Pugh class B patients, all C‐indices were near 0.50; mALBI‐T was 0.496 (95% CI, 0.428–0.571). The mALBI‐T score‐by‐Child‐Pugh class interaction was significant (*p* = 0.036), suggesting that the score‐mortality association differed by Child‐Pugh class.

**TABLE 5:** Overall survival performance in Child‐Pugh class subgroups.

Child‐Pugh subgroup	Model	AIC/N	Harrell C‐index (95% CI)
A (*n* = 831; deaths = 338)	CP‐T	4.90	0.559 (0.529–0.590)
A (*n* = 831; deaths = 338)	ALBI‐T	4.86	0.602 (0.572–0.632)
A (*n* = 831; deaths = 338)	mALBI‐T	4.84	0.619 (0.589–0.649)
A (*n* = 831; deaths = 338)	pALBI‐T	4.87	0.600 (0.568–0.632)
B (*n* = 119; deaths = 79)	CP‐T	5.22	0.489 (0.411–0.566)
B (*n* = 119; deaths = 79)	ALBI‐T	5.25	0.497 (0.428–0.572)
B (*n* = 119; deaths = 79)	mALBI‐T	5.25	0.496 (0.428–0.571)
B (*n* = 119; deaths = 79)	pALBI‐T	5.26	0.485 (0.413–0.559)

*Note:* AIC/N is AIC divided by the number of patients in the subgroup; model fit was compared within each subgroup.

Abbreviations: AIC, Akaike information criterion; ALBI‐T, albumin‐bilirubin‐tumor; CI, confidence interval; CP‐T, Child‐Pugh‐tumor; mALBI‐T, modified albumin‐bilirubin‐tumor; pALBI‐T, platelet‐albumin‐bilirubin‐tumor.

mALBI‐T ranked first in both eras (C‐index 0.607 in 2002–2009 and 0.635 in 2010–2016; interaction *p* = 0.522) and across hepatitis B virus (HBV), hepatitis C virus (HCV), HBV/HCV coinfection, and non‐B/non‐C etiologies (interaction *p* = 0.961) (Supplementary Table 1). It also ranked first in women (0.660) and men (0.608). The score‐by‐sex interaction was significant (*p* = 0.025), suggesting that the association between mALBI‐T and mortality differed by sex; this exploratory finding requires independent validation.

### 3.5 Recurrence and Exploratory Comparisons

There were 598 recurrences and 723 RFS events. mALBI‐T had the lowest RFS AIC (8769.7) and highest C‐index (0.599; 95% CI, 0.578–0.620) (Supplementary Table 2). Differences were significant versus CP‐T (*p* = 0.006) and pALBI‐T (*p* = 0.047), but not ALBI‐T (*p* = 0.102). Repeat RFA (344/598, 57.5%) and transarterial embolization or chemoembolization (178/598, 29.8%) were the most frequent first salvage treatments.

Among 756 complete cases, PNI and GNRI had numerically higher C‐indices than mALBI‐T (0.629 and 0.632 vs. 0.613), but paired differences were not significant (*p* = 0.228 and *p* = 0.110); GNRI had the lowest AIC (Supplementary Table 3). NLR performed worse than mALBI‐T (*p* = 0.027). In all 950 patients, the refitted Kao‐variable model had a similar C‐index (0.627 vs. 0.628; difference, 0.001; *p* = 0.950) but higher AIC (5067.0 vs. 5052.2).

## 4 Discussion

In the present study, mALBI‐T showed the best prognostic performance among the four combined hepatic reserve and tumor‐stage models in patients with BCLC Stages 0 or A HCC who received RFA as initial therapy and achieved complete ablation. mALBI‐T had the lowest AIC and highest Harrell C‐index. Paired bootstrap comparisons supported higher discrimination than CP‐T, ALBI‐T, and pALBI‐T, although the absolute differences were modest and overall discrimination remained moderate. Apparent calibration was generally close. Its advantage was most evident at 5 years and in Child‐Pugh Class A patients, whereas all models showed limited discrimination in the Child‐Pugh Class B subgroup.

For the hepatic reserve component, the use of an objective albumin‐bilirubin‐based assessment of liver function is consistent with RFA studies showing that ALBI grade predicts recurrence and prognosis after ablation [28, 29]. Ho et al. evaluated 499 patients undergoing RFA and found ALBI to be the best‐performing noninvasive liver‐function model [15]. Chen et al. found that ALBI Grades 2 or 3 independently predicted poorer OS and progression‐free survival in 271 patients with solitary HCC < 2 cm [17]. In HBV‐associated HCC, Chan et al. also showed that ALBI‐T performed better than the Japan Integrated Staging score [30].

The added value of mALBI lies in its ability to separate the broad ALBI Grade 2 category into two subgrades, thereby identifying differences in hepatic reserve that are not captured by Child‐Pugh class or conventional ALBI grade. This distinction is particularly relevant after complete ablation, when liver decompensation, liver failure, development of new tumors, and tolerance of salvage therapy might all affect long‐term survival. In our cohort, 500 patients (52.6%) were ALBI Grade 2; mALBI classified 207 (21.8%) as Grade 2a and 293 (30.8%) as Grade 2b. This additional stratification may explain why mALBI‐T consistently outperformed ALBI‐T.

For the tumor component, AJCC eighth edition T stage complements hepatic‐reserve grading by distinguishing solitary tumors ≤ 2 cm, larger solitary tumors, and multiple tumors. It performed best among the candidate tumor‐stage systems, and its Harrell C‐index increased from 0.552 to 0.628 when combined with mALBI. These findings support the inclusion of both hepatic reserve and tumor burden in prognostic assessment after RFA.

Recent post‐RFA recurrence data also support this dual‐axis approach. Wang et al. found that Child‐Pugh class and tumor factors independently predicted intrahepatic recurrence‐free survival after RFA [31]. In the present study, mALBI‐T also had the best fit and discrimination for RFS. Differences were significant compared with CP‐T and pALBI‐T, but not ALBI‐T, and discrimination remained modest. Repeat RFA and transarterial embolization or chemoembolization were the most frequent first salvage treatments.

These findings are compatible with previous studies. In a multicenter Japanese cohort, mALBI‐T showed better prognostic performance than ALBI‐T and the Japan Integrated Staging score [22], and an Egyptian validation study also reported favorable performance [26]. The present study extends these findings to a homogeneous cohort restricted to BCLC Stages 0 or A HCC, RFA as initial therapy, and complete ablation, using AJCC eighth edition T stage as the tumor component.

Time‐dependent AUROC analysis showed different patterns over time. pALBI‐T had the highest 1‐year AUROC, possibly because platelet count reflects portal hypertension and fibrosis. mALBI‐T had the highest 5‐year AUROC, whereas mALBI‐T and pALBI‐T performed similarly at 10 years. Thus, mALBI‐T had the highest overall discrimination and 5‐year AUROC, but no single model was superior at every time point.

The subgroup analysis showed that mALBI‐T was most useful in Child‐Pugh class A patients, who are the main candidates for curative‐intent RFA. In Child‐Pugh class B patients, all models had C‐index values close to 0.5. This may reflect restricted selection for ablation and greater influence of liver‐related mortality, although cause‐specific death was unavailable and the small subgroup limits precision. mALBI‐T remained the top‐ranked score across diagnosis eras and etiologies. The stronger score‐mortality association observed in women was exploratory and requires independent confirmation.

Comparison with other prognostic approaches further clarifies the role of mALBI‐T. Kao et al. reported a bootstrap‐corrected C‐index of 0.770 for an ALBI‐based nomogram in 622 patients within Milan criteria [27]. A locally refitted model using those five predictors had discrimination similar to mALBI‐T, whereas mALBI‐T had lower AIC and used fewer input variables. This exploratory comparison does not establish superiority over the published nomogram. No statistically significant differences in discrimination were detected between mALBI‐T and PNI or GNRI in the complete‐case analysis, and while these markers could also aid prognostic assessment in very early HCC [32]. mALBI‐T offers a simple approach alongside these models.

mALBI‐T uses routine laboratory data and AJCC eighth edition T stage and can be calculated without specialized testing. It could support prognostic counseling and multidisciplinary assessment after complete ablation, although thresholds for changing surveillance or retreatment have not yet been established.

The strengths of this study include its large, homogeneous cohort, complete initial ablation, long follow‐up, and assessment of model fit, discrimination and calibration, although as this was a retrospective single‐center study of predominantly viral hepatitis‐related HCC treated during 2002–2016, criterion‐specific exclusion counts, procedure‐level device use, monocyte counts, ablative margins, cause‐specific death and complete subsequent treatment sequences were unavailable, while the Child‐Pugh class B and biomarker analyses had smaller samples. Tumor‐stage selection and calibration used the same cohort, which might have produced optimistic estimates. Although RFS and first salvage therapy were analyzed, repeated recurrence and later treatments were not modeled. Prospective external validation should include contemporary, etiologically diverse cohorts, dedicated Child‐Pugh class B evaluation, and longitudinal recurrence and treatment data.

Among CP‐T, ALBI‐T, mALBI‐T, and pALBI‐T, mALBI‐T provided the best prognostic stratification for OS in patients with BCLC Stages 0 or A HCC treated with RFA achieving complete ablation. Its advantage was most evident in overall discrimination, 5‐year prediction, and Child‐Pugh Class A patients. By combining objective mALBI‐based hepatic reserve assessment with AJCC eighth edition T stage, mALBI‐T is a simple and clinically applicable tool for prognostic assessment after RFA in early HCC. Given its moderate discrimination, it should complement individualized clinical assessment.

## Conflicts of Interest

The authors declare no conflicts of interest.

## Supporting information


**Supplementary Figure 1.** Apparent calibration of all four total scoring systems. Cox‐predicted and Kaplan–Meier‐observed survival probabilities are shown at 1, 5, and 10 years by score category. ALBI‐T, albumin‐bilirubin‐tumor; CP‐T, Child‐Pugh‐tumor; mALBI‐T, modified albumin‐bilirubin‐tumor; OS, overall survival; pALBI‐T, platelet‐albumin‐bilirubin‐tumor.
**Supplementary Table 1**. Overall survival discrimination by diagnosis era, sex, and etiology.
**Supplementary Table 2A**. Recurrence‐free survival performance.
**Supplementary Table 2B**. First salvage treatment among 598 patients with recurrence.
**Supplementary Table 3A**. Complete‐case comparison with nutrition‐ and inflammation‐based biomarkers (*n* = 756).
**Supplementary Table 3B**. Comparison with a locally refitted Kao‐variable model.

## Data Availability

The data that support the findings of this study are available on request from the corresponding author. The data are not publicly available due to privacy or ethical restrictions.
