# Supplementary material for: Validation of Modified Albumin‐Bilirubin‐Tumor Stage as a Prognostic Model in Early‐Stage Hepatocellular Carcinoma Treated With Radiofrequency Ablation
Source: Kaohsiung J Med Sci. 2026 Nov 2:e70313. Online ahead of print. doi: 10.1002/kjm2.70313 (PMC13728425; doi:10.1002/kjm2.70313)
Supplement: Supplementary file 1 [file KJM2-9999-e70313-s001.docx]

**Supplementary material**

Validation of modified albumin-bilirubin-tumor stage as a prognostic model in early-stage hepatocellular carcinoma treated with radiofrequency ablation

**Supplementary methods**

The archived follow-up labels 'dead' (374 patients) and 'critical AAD' (43 patients) both denoted death and were included among the 417 overall survival events.

The prognostic nutritional index was calculated as 10 x albumin (g/dL) + 0.005 x absolute lymphocyte count (/mm3). The geriatric nutritional risk index was calculated as 1.489 x albumin (g/L) + 41.7 x actual/ideal body weight, with the weight ratio capped at 1 and ideal weight defined as 22 x height (m)^2. Neutrophil-to-lymphocyte ratio was calculated from differential counts. Biomarker comparisons used 756 common complete cases; 194 patients missing one or more required inputs were excluded, and no values were imputed. The locally refitted Kao-variable model included age >65 years, international normalized ratio >1.1, alpha-fetoprotein >20 ng/mL, multiple tumors, and albumin-bilirubin grade 2/3.

| **Supplementary Table 1. Overall survival discrimination by diagnosis era, sex, and etiology** | | | |
| --- | --- | --- | --- |
| **Stratifier** | **Subgroup** | **Model** | **Harrell C-index (95% CI)** |
| Diagnosis era | 2002-2009 (n = 357; events = 247) | CP-T | 0.565 (0.529-0.600) |
| Diagnosis era | 2002-2009 (n = 357; events = 247) | ALBI-T | 0.583 (0.548-0.619) |
| Diagnosis era | 2002-2009 (n = 357; events = 247) | mALBI-T | 0.607 (0.571-0.644) |
| Diagnosis era | 2002-2009 (n = 357; events = 247) | pALBI-T | 0.584 (0.547-0.621) |
| Diagnosis era | 2010-2016 (n = 593; events = 170) | CP-T | 0.586 (0.548-0.626) |
| Diagnosis era | 2010-2016 (n = 593; events = 170) | ALBI-T | 0.618 (0.579-0.657) |
| Diagnosis era | 2010-2016 (n = 593; events = 170) | mALBI-T | 0.635 (0.594-0.674) |
| Diagnosis era | 2010-2016 (n = 593; events = 170) | pALBI-T | 0.619 (0.580-0.659) |
| Sex | Female (n = 359; events = 155) | CP-T | 0.620 (0.581-0.661) |
| Sex | Female (n = 359; events = 155) | ALBI-T | 0.637 (0.594-0.681) |
| Sex | Female (n = 359; events = 155) | mALBI-T | 0.660 (0.616-0.704) |
| Sex | Female (n = 359; events = 155) | pALBI-T | 0.658 (0.614-0.700) |
| Sex | Male (n = 591; events = 262) | CP-T | 0.551 (0.517-0.585) |
| Sex | Male (n = 591; events = 262) | ALBI-T | 0.587 (0.553-0.619) |
| Sex | Male (n = 591; events = 262) | mALBI-T | 0.608 (0.572-0.642) |
| Sex | Male (n = 591; events = 262) | pALBI-T | 0.578 (0.541-0.614) |
| Etiology | HBV (n = 326; events = 126) | CP-T | 0.587 (0.540-0.634) |
| Etiology | HBV (n = 326; events = 126) | ALBI-T | 0.619 (0.576-0.663) |
| Etiology | HBV (n = 326; events = 126) | mALBI-T | 0.636 (0.591-0.680) |
| Etiology | HBV (n = 326; events = 126) | pALBI-T | 0.614 (0.573-0.660) |
| Etiology | HBV/HCV (n = 67; events = 33) | CP-T | 0.558 (0.467-0.654) |
| Etiology | HBV/HCV (n = 67; events = 33) | ALBI-T | 0.561 (0.478-0.662) |
| Etiology | HBV/HCV (n = 67; events = 33) | mALBI-T | 0.635 (0.554-0.726) |
| Etiology | HBV/HCV (n = 67; events = 33) | pALBI-T | 0.615 (0.530-0.715) |
| Etiology | HCV (n = 450; events = 210) | CP-T | 0.574 (0.534-0.615) |
| Etiology | HCV (n = 450; events = 210) | ALBI-T | 0.606 (0.565-0.646) |
| Etiology | HCV (n = 450; events = 210) | mALBI-T | 0.623 (0.583-0.664) |
| Etiology | HCV (n = 450; events = 210) | pALBI-T | 0.606 (0.565-0.649) |
| Etiology | Non-B/non-C (n = 107; events = 48) | CP-T | 0.570 (0.493-0.653) |
| Etiology | Non-B/non-C (n = 107; events = 48) | ALBI-T | 0.591 (0.504-0.672) |
| Etiology | Non-B/non-C (n = 107; events = 48) | mALBI-T | 0.608 (0.519-0.693) |
| Etiology | Non-B/non-C (n = 107; events = 48) | pALBI-T | 0.605 (0.520-0.693) |
| Interaction p values for mALBI-T were 0.522 for era, 0.025 for sex, and 0.961 for etiology. These Cox interaction tests evaluate differences in the score-mortality association, not direct differences between subgroup C-indices. ALBI-T, albumin-bilirubin-tumor; CI, confidence interval; CP-T, Child-Pugh-tumor; HBV, hepatitis B virus; HCV, hepatitis C virus; mALBI-T, modified albumin-bilirubin-tumor; pALBI-T, platelet-albumin-bilirubin-tumor. | | | |

| **Supplementary Table 2A. Recurrence-free survival performance** | | | | | |
| --- | --- | --- | --- | --- | --- |
| **Model** | **AIC** | **Delta AIC** | **Harrell C-index (95% CI)** | **mALBI-T minus comparator (95% CI)** | **p value** |
| CP-T | 8793.7 | 24.1 | 0.578 (0.558-0.600) | 0.021 (0.006-0.036) | 0.006 |
| ALBI-T | 8773.9 | 4.2 | 0.593 (0.572-0.614) | 0.006 (-0.001-0.014) | 0.102 |
| mALBI-T | 8769.7 | 0.0 | 0.599 (0.578-0.620) | Reference | - |
| pALBI-T | 8784.2 | 14.6 | 0.587 (0.566-0.609) | 0.012 (0.000-0.024) | 0.047 |
| Recurrence-free survival was measured from initial radiofrequency ablation to first recurrence or death. AIC, Akaike information criterion; ALBI-T, albumin-bilirubin-tumor; CI, confidence interval; CP-T, Child-Pugh-tumor; mALBI-T, modified albumin-bilirubin-tumor; pALBI-T, platelet-albumin-bilirubin-tumor. | | | | | |

| **Supplementary Table 2B. First salvage treatment among 598 patients with recurrence** | | |
| --- | --- | --- |
| **First salvage treatment** | **n** | **%** |
| Repeat RFA | 344 | 57.5 |
| Transarterial embolization/chemoembolization | 178 | 29.8 |
| Percutaneous ethanol injection | 37 | 6.2 |
| Missing/not recorded | 13 | 2.2 |
| Surgical resection | 11 | 1.8 |
| Radiotherapy | 6 | 1.0 |
| Targeted therapy | 4 | 0.7 |
| Chemotherapy | 2 | 0.3 |
| No treatment | 2 | 0.3 |
| Multiple modalities | 1 | 0.2 |
| RFA, radiofrequency ablation. | | |

| **Supplementary Table 3A. Complete-case comparison with nutrition- and inflammation-based biomarkers (n = 756)** | | | | |
| --- | --- | --- | --- | --- |
| **Model** | **AIC** | **Harrell C-index (95% CI)** | **mALBI-T minus comparator (95% CI)** | **p value** |
| mALBI-T | 3866.9 | 0.613 (0.581-0.643) | Reference | - |
| PNI (continuous) | 3868.4 | 0.629 (0.598-0.659) | -0.016 (-0.041-0.011) | 0.228 |
| GNRI (continuous) | 3862.0 | 0.632 (0.601-0.663) | -0.019 (-0.042-0.004) | 0.110 |
| NLR (continuous) | 3924.0 | 0.561 (0.528-0.596) | 0.052 (0.007-0.097) | 0.027 |
| PNI <45 | 3881.2 | 0.594 (0.566-0.621) | 0.019 (-0.008-0.046) | 0.150 |
| AIC, Akaike information criterion; CI, confidence interval; GNRI, geriatric nutritional risk index; mALBI-T, modified albumin-bilirubin-tumor; NLR, neutrophil-to-lymphocyte ratio; PNI, prognostic nutritional index. | | | | |

| **Supplementary Table 3B. Comparison with a locally refitted Kao-variable model** | | | | |
| --- | --- | --- | --- | --- |
| **Model** | **AIC** | **Harrell C-index (95% CI)** | **mALBI-T minus comparator (95% CI)** | **p value** |
| mALBI-T | 5052.2 | 0.628 (0.601-0.655) | Reference | - |
| Locally refitted Kao-variable model | 5067.0 | 0.627 (0.598-0.655) | 0.001 (-0.021-0.022) | 0.950 |
| The locally refitted model used age >65 years, international normalized ratio >1.1, alpha-fetoprotein >20 ng/mL, multiple tumors, and albumin-bilirubin grade 2/3. This is not external validation of the published nomogram. The marginal mALBI-T confidence interval is reproduced from Table 3; the paired difference uses the comparison-specific bootstrap. AIC, Akaike information criterion; CI, confidence interval; mALBI-T, modified albumin-bilirubin-tumor. | | | | |

**Figure S1. Apparent calibration of all total scoring systems**
